# The Role of Dietary Extra Virgin Olive Oil and Corn Oil on the Alteration of Epigenetic Patterns in the Rat DMBA-Induced Breast Cancer Model

**DOI:** 10.1371/journal.pone.0138980

**Published:** 2015-09-24

**Authors:** Cristina Rodríguez-Miguel, Raquel Moral, Raquel Escrich, Elena Vela, Montserrat Solanas, Eduard Escrich

**Affiliations:** Grup Multidisciplinari per a l’Estudi del Càncer de Mama, Physiology Unit, Department of Cell Biology, Physiology and Immunology, Faculty of Medicine, Universitat Autònoma de Barcelona, Bellaterra, Barcelona, Spain; University of North Carolina School of Medicine, UNITED STATES

## Abstract

Disruption of epigenetic patterns is a major change occurring in all types of cancers. Such alterations are characterized by global DNA hypomethylation, gene-promoter hypermethylation and aberrant histone modifications, and may be modified by environment. Nutritional factors, and especially dietary lipids, have a role in the etiology of breast cancer. Thus, we aimed to analyze the influence of different high fat diets on DNA methylation and histone modifications in the rat dimethylbenz(a)anthracene (DMBA)-induced breast cancer model. Female Sprague-Dawley rats were fed a low-fat, a high corn-oil or a high extra-virgin olive oil (EVOO) diet from weaning or from induction with DMBA. In mammary glands and tumors we analyzed global and gene specific (*RASSF1A*, *TIMP3*) DNA methylation by LUMA and bisulfite pyrosequencing assays, respectively. We also determined gene expression and enzymatic activity of DNA methyltransferases (*DNMT1*, *DNMT3a* and *DNMT3b*) and evaluated changes in histone modifications (H3K4me2, H3K27me3, H4K20me3 and H4K16ac) by western-blot. Our results showed variations along time in the global DNA methylation of the mammary gland displaying decreases at puberty and with aging. The olive oil-enriched diet, on the one hand, increased the levels of global DNA methylation in mammary gland and tumor, and on the other, changed histone modifications patterns. The corn oil-enriched diet increased DNA methyltransferase activity in both tissues, resulting in an increase in the promoter methylation of the tumor suppressor genes *RASSF1A* and *TIMP3*. These results suggest a differential effect of the high fat diets on epigenetic patterns with a relevant role in the neoplastic transformation, which could be one of the mechanisms of their differential promoter effect, clearly stimulating for the high corn-oil diet and with a weaker influence for the high EVOO diet, on breast cancer progression.

## Introduction

Breast cancer is the most frequent malignant neoplasia among women worldwide [1]. In addition to genetic, epigenetic and endocrine factors, the environment, and specifically nutritional factors, plays a key role in its etiology. Epidemiological evidence and research studies in animal models suggest that diet, and mainly dietary lipids, play an important role in breast cancer development [2]. Thus, n-6 polyunsaturated fatty acids (PUFA) from vegetable oils, especially linoleic acid (18:2n-6) and saturated fat, mainly from animal origin, have shown a stimulating effect on breast cancer. In contrast, an inhibitory effect has been described for n-3 PUFA, conjugated linoleic acid and γ-linolenic acid. Monounsaturated fatty acids (MUFA), mainly oleic acid (18:1n-9, OA), present in high quantities in olive oil, seems to be protective, although some inconsistent data have been reported ranging from protective to weak stimulating effects on tumor growth [3, 4]. In this sense, abundant results have attributed a protective effect on breast cancer risk to Mediterranean Diet, characterized by the consumption of olive oil as the main source of energy [5]. The specific mechanisms by which EVOO (Extra Virgin Olive Oil) and other dietary lipids may exert their modulatory effects on cancer are not fully understood.

Several nutrients with anticancer potential have shown to influence epigenome by interfering with processes deregulated during carcinogenesis, such as global DNA hypomethylation, tumor suppressor gene promoter hypermethylation and aberrant histone modifications [6]. Global DNA hypomethylation reflects loss of methylation associated with aberrant expression of some genes that could contribute to neoplastic transformation, tumorigenesis, cancer progression and chromosomal instability [7]. On the other hand, methylation-dependent gene silencing is a normal mechanism for regulation of gene expression [8]. In cancer cells, gene promoter hypermethylation represents a mutation-independent mechanism for inactivation of tumor suppressor genes. A significant number of cancer-related genes are subject to methylation-dependent silencing, and many of these genes contribute to the hallmarks of cancer, such as *RASSF1A* (Ras-association domain family 1, isoform A) and *TIMP3* (Tissue inhibitor of metalloproteinase-3) [9]. DNA methylation is catalyzed by the enzyme 5-cytosine DNA methyltransferase (DNMT) commonly classified as maintenance (*DNMT1*) and *de novo* (*DNMT3a* and *DNMT3b*). The levels of DNMT, especially those of *DNMT3a* and *DNMT3b*, are often increased in various cancer tissues and cell lines. This may partially account for the hypermethylation of promoter CpG-rich regions of tumor suppressor genes in a variety of malignancies [10]. In addition to DNA methylation disruption, aberrant histone modifications play a role in the carcinogenesis process. Post-translational modifications of histone determine DNA-histone interaction and transcriptional activity of genome, having a functional cross-talk with DNA methylation. Among the various modifications, acetylation and methylation of histone lysines such as H4K16ac, H4K20me3, H3K27me3 and H3K4me2, have been frequently associated with breast cancer [11–13].

Given the increasingly evidence that dietary factors can influence epigenetic changes, a better knowledge of the interrelationships among dietary lipids, epigenetic modifications and breast cancer is necessary to determine the utility of interventions with nutritional components for breast cancer prevention. Hence, the aim of the present study is to analyze the influence of different high fat diets, rich in corn oil or in EVOO, on DNA methylation and histone modifications in the rat dimethylbenz(a)anthracene-induced breast cancer model.

## Materials and Methods

### Animals and experimental design

All animals received humane care under an institutionally approved experimental animal protocol, following the legislation applicable in this country. The protocol, which included euthanasia by decapitation, was approved by the Ethical Committee of Animal and Human Experimentation of our institution (CEEAH 566/3616). Female Sprague-Dawley rats were obtained from Charles River Lab. (L’Arbresle, France, N = 167) and housed 2–3 per cage in a controlled environment and 12:12h light/dark cycle. The day after arrival (23 days of age), 6 animals were euthanized by decapitation and the remaining rats distributed upon the type of diet and timing of dietary intervention (S1 Fig). Three semi-synthetic diets were designed: a low fat diet (3% corn oil-w/w-), a high corn oil diet (20% corn oil) and a high olive oil diet (3% corn oil + 17% extra virgin olive oil). The composition, preparation and suitability of the experimental diets have been described previously [14–16]. Thus, from weaning onwards, control animals were fed the low fat diet (Group LF, N = 87), while the high fat groups animals were fed the high corn oil diet (Group HCO, N = 37) or the high extra virgin olive oil diet (Group HOO, N = 37) and water ad libitum. At 53 days of age, mammary cancer was induced by oral gavage with one single dose of 5 mg of dimethylbenz(a)anthracene (DMBA) (Sigma-Aldrich; St. Louis, MO, USA) dissolved in corn oil. To study the promotion of the carcinogenesis, after DMBA treatment 50 rats from the LF group were changed to high fat dietary intervention, thus forming the Group low fat—high corn oil (LF-HCO, N = 25), and the Group low fat—high extra virgin olive oil (LF-HOO, N = 25). Animals were euthanized at day 36 (N = 6 / experimental condition: control, HCO, HOO), 51 (N = 6 / experimental condition: control, HCO, HOO), 100 (N = 6 / group) and 236–256 (median 246 days, end of the assay), (N = 20 / group). Abdominal mammary glands were collected and flash frozen for molecular analyses. At the end of the assay tumors were excised, a portion fixed in 4% formalin for histopathological diagnosis [17], and the rest flash frozen for molecular analyses. Only data from confirmed mammary adenocarcinomas has been included in this study.

### DNA isolation and bisulfite modification

Genomic DNA was extracted from mammary glands and tumors using the SpeedTools Tissue DNA Extraction kit (Biotools B&M Labs, Madrid, Spain), according to the manufacturer's recommendations. The concentration of extracted DNAs was determined using the NanoDrop ND-1000 spectrophotometer (Thermo Fisher Scientific). The 260/280 and 260/230 nm ratios were used to evaluate the DNA purity and the integrity was assessed by 1% agarose gel electrophoresis and ethidium bromide staining.

The extracted DNAs were treated with sodium bisulfite using the CpGenome™ Turbo Bisulfite Modification Kit (Merck Millipore, Billerica, MA, USA), converting all unmethylated cytosine to uracil.

### Determination of global DNA methylation level (LUminometric Methylation Assay-LUMA-)

Global DNA methylation level was determined by LUMA as described previously [18] with modifications [19]. Briefly, genomic DNA was cleaved with *Hpa*II + *Eco*RI-HF or *Msp*I + *Eco*RI-HF (New England Biolabs) in two parallel reactions containing 500 ng genomic DNA and 5U of each restriction enzyme. The reactions were incubated for 4 hours at 37°C. Samples were analyzed in the Centre for Research in Agricultural Genomics (CRAG) from the Universitat Autònoma de Barcelona, using the PyroMark Q96 ID System (Qiagen), with the following dispensation order: GTGTCACATGTGTG. Percentage of DNA methylation was expressed as [1 –(*Hpa*II+*EcoRI* ΣG/ΣT/(*MspI+EcoRI* ΣG/ΣT)]*100. This percentage represents the amount of 5-mC within the CCGG motif throughout the genome.

### RNA isolation and gene expression analysis by Real-Time PCR

Total RNA from mammary glands and tumors were extracted using the Tissue RNeasy Extraction Kit (Qiagen, Hilden, Germany). RNA was quantified spectrophotometrically with NanoDrop ND-1000 spectrophotometer (Thermo Fisher Scientific, Waltham, MA, USA), and integrity was assessed by 2% agarose gel electrophoresis and ethidium bromide staining. Two micrograms of total RNA were reverse transcribed using the High Capacity cDNA Reverse Transcription Kit (Applied Biosystems, Foster City, CA, USA). The study of expression of specific genes was performed by real-time PCR using the TaqMan methodology (Applied Biosystems), in the iCycler MyiQ Real-Time PCR detection system (Bio-Rad Laboratories, Hercules, CA, USA). Reactions were prepared with the TaqMan Universal PCR Master Mix and the suitable TaqMan assay: Rn01445298_m1 (*RASSF1A*), Rn00441826_m1 (*TIMP3*), Rn00709664_m1 (*DNMT1*), Rn01469994_g1 (*DNMT3A*) and Rn01536419_m1 (*DNMT3B*). Twenty nanograms of cDNA were amplified during 40 cycles of 15 seconds at 95°C and 60 seconds at 60°C. Gene expression was normalized using *HPRT1* (Rn01527840_m1) as a control transcript.

### Quantitative methylation analysis (pyrosequencing)

Bisulfite pyrosequencing was used to determine the promoter methylation of *RASSF1A* and *TIMP3* genes in bisulfite-modified DNAs, from mammary glands and tumors. Predesigned methylation assays were used to determine the methylation status of three and six CpG sites in the *RASSF1A* (PM00416297) and *TIMP3* (PM00574896) promoter respectively (PyroMark CpG assay, Qiagen). Briefly, 1 μl of bisulfite-modified DNA (25 ng) was amplified using 1U Platinum® Taq High Fidelity (Thermo Fisher Scientific) in a 25 μl final volume. The amplification conditions were: denaturating at 95°C for 5 minutes, followed by 45 cycles at 95°C for 30 seconds, at 55°C for 30 seconds, at 72°C for 45 seconds and a final extension at 72°C for 5 minutes. Simple robust amplification was confirmed by visualization in 2% agarose gel stained with ethidium bromide. Pyrosequencing reactions were then analyzed in the Centre for Research in Agricultural Genomics (CRAG) from the Universitat Autònoma de Barcelona, using in the PSQ HS 96 Pyrosequencing Instrument. Methylation data were presented as the percentage of average methylation in all observed CpG sites.

### DNMT activity

Global DNA methyltransferase (DNMT) activity was evaluated in nuclear protein extracts from mammary glands and tumors obtained using the EpiQuik Nuclear Extraction Kit (Epigentek, Farmingdale, NY, USA). DNA methyltransferase activity was measured in duplicates using the EpiQuik™ DNMTActivity/Inhibition Assay Ultra Kit (Epigentek), following manufacturer’s recommendations. Replicates of each sample (include blank and positive control) were analyzed to validate the signal generated. The DNMT activity data were presented as OD/h/mg.

### Histone modifications

Modifications of histone 4 (H4K20me3, H4K16ac) and histone 3 (H3K4me2, H3K27me3) were determined in nuclear protein extracts from mammary glands and tumors. Samples were resolved by SDS-PAGE in Any kD™ Mini-PROTEAN® TGX Stain-Free™ Gels (Bio-Rad Laboratories) for 30 minutes at 200 volts, and transferred to PVDF membranes with Trans-Blot® Turbo™ Transfer System (Bio-Rad Laboratories). Primary antibodies used were Anti-Histone H4 (tri methyl K20) (1:5000), Anti-Histone H4 (acetyl K16) (1:5000), Anti-Histone H4 (1:5000), Anti-Histone H3 (di methyl K4) (1:1000), Anti-Histone H3 (1:10000) from Abcam (Cambrigde, UK) and Anti-Histone H3 (tri methyl K27) (1:1000) from Epigentek (Farmingdale, NY, USA). Horseradish peroxidase conjugated rabbit secondary antibody was obtained from Sigma–Aldrich (St. Louis, MO, USA). Immunoreactive proteins were visualized using the Luminata™ Forte Western HRP Substrate (Merck Millipore). Densitometric values of bands were analyzed and normalized to the protein loaded on each sample using the ChemiDoc XRS+ system and the Image Lab™ software (Bio-Rad Laboratories). Values were related to an internal control pool loaded in duplicate in each gel, and normalized to global histone 3 or histone 4.

### Statistical analysis

Statistical analyses were performed using SPSS software (20.0 Version) and R/Deducer software (3.1.2 Version). The distribution of each variable was determined by the Kolmogoroz–Smirnov test and the equality of variances among groups was determined by Leven’s test. Quantitative data were analyzed by the nonparametric Mann–Whitney’s U-test. The level of significance was established at P<0.05.

## Results

### Effects of high fat diets and breast cancer on global DNA methylation

**Fig 1:**
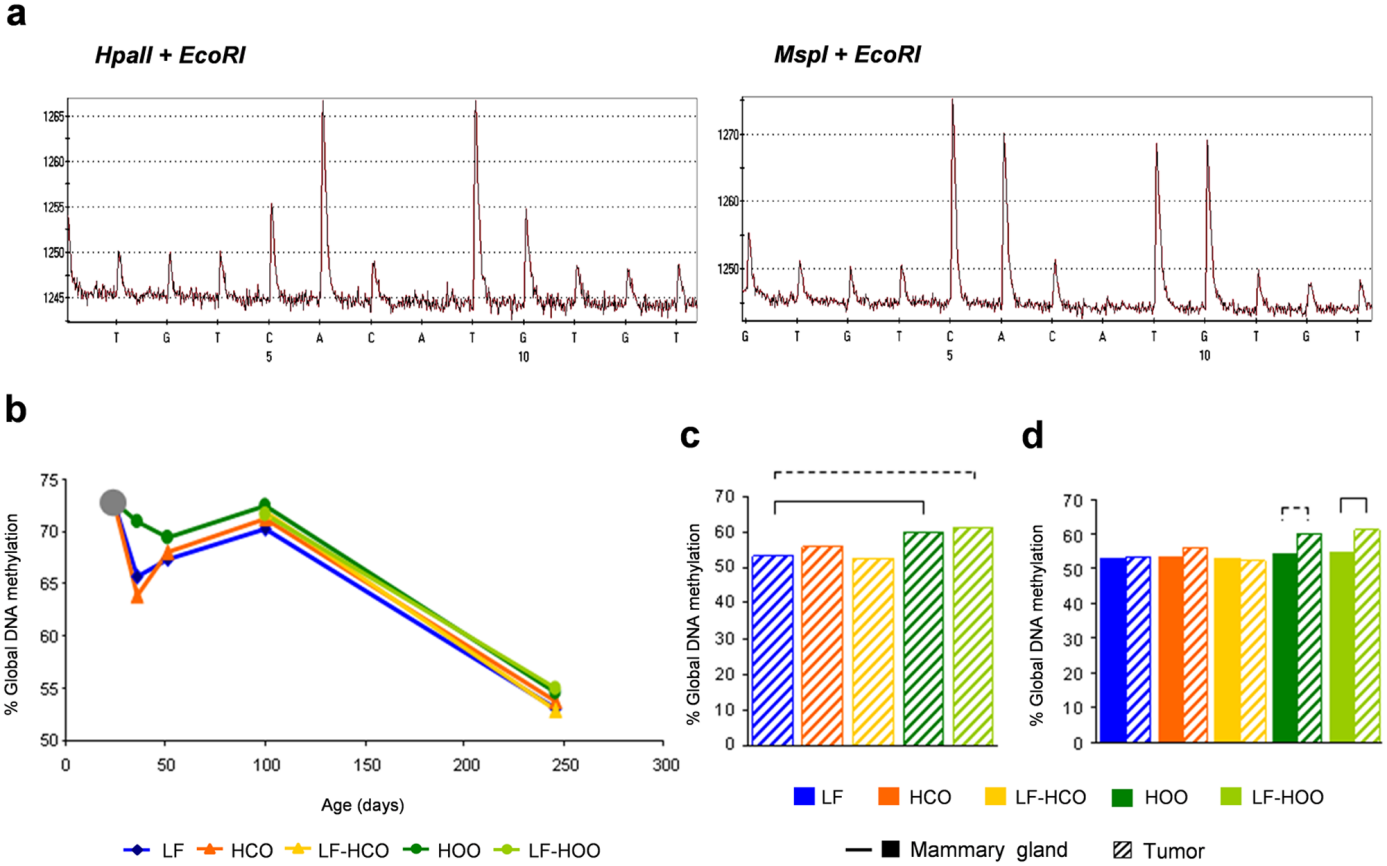
Global DNA methylation level in mammary gland and tumor. **(a)** Representative pyrograms obtained in LUMA assay of DNA cleaved with *HpaII*+*EcoRI-HF* and *MspI*+*EcoRI-HF*. **(b)** Determination of global DNA methylation in mammary gland from all experimental groups along time. **(c)** Global DNA methylation in tumors from all experimental groups at the end of the assay (246 days). **(d)** Comparison of global DNA methylation between mammary gland and tumors in each experimental group at 246 days of age. Data shown represent medians of the groups. Full lines connecting groups indicate differences statistically significant (p<0.05); dotted lines indicate differences close to significance (p<0.1).

Results of global DNA methylation in mammary gland displayed variations along time, showing clear decreases at puberty (from 23 to 36 days) and in adulthood (from 100 to 246 days) (Fig 1b). In relation to the effect of diets, the group fed with the olive oil one (HOO) showed the highest methylation levels at all ages, close to significance compared to HCO at 36 days and compared to LF at 51 days of age (Fig 1b). In mammary tumors, both groups fed the olive oil-enriched diet had higher values of global DNA methylation (significantly in HOO and close to significance in LF-HOO) compared to LF group (Fig 1c). Finally, we compared the global DNA methylation between mammary gland and tumor in each experimental group at 246 days of age, finding no clear differences among tissues, except an increase in tumor compared to mammary gland in both groups fed the olive oil-enriched diet (significantly in LF-HOO and close to significance in HOO) (Fig 1d).

### Effects of high fat diets and breast cancer on RASSF1A and TIMP3 mRNA relative levels

**Fig 2:**
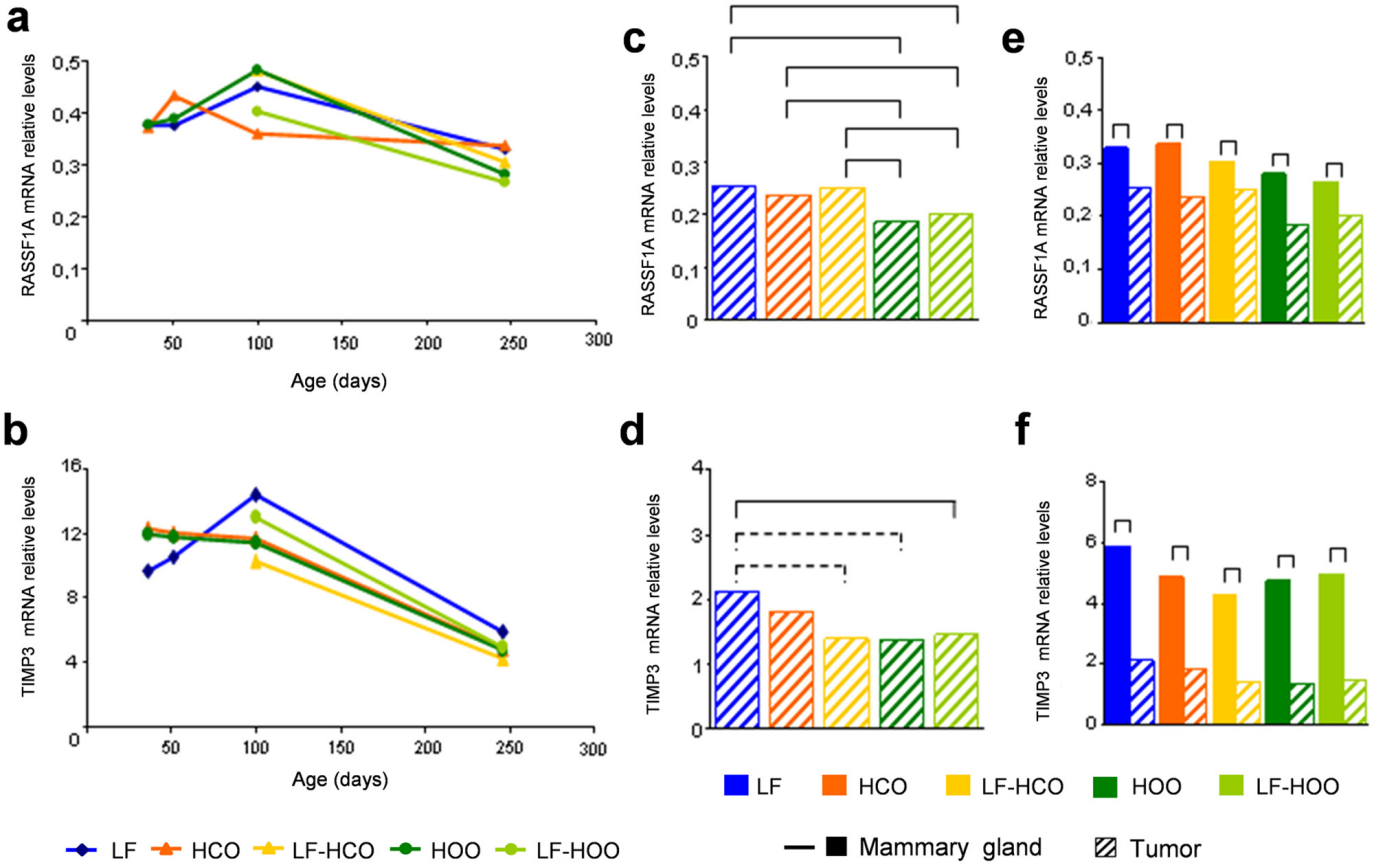
*RASSF1A* and *TIMP3* gene expression in mammary gland and tumor. Determination of *RASSF1A* **(a)** and *TIMP3* **(b)** mRNA relative levels in mammary gland from all experimental groups along time. Determination of *RASSF1A* **(c)** and *TIMP3* **(d)** mRNA relative levels in tumors from all experimental groups at 246 days of age. Comparison of *RASSF1A* **(e)** and *TIMP3* **(f)** mRNA relative levels between both tissues in each experimental group at 246 days of age. Data shown represent medians of the groups. Full lines connecting groups indicate differences statistically significant (p<0.05); dotted lines indicate differences close to significance (p<0.1).

Results for *RASSF1A* and *TIMP3* gene expression in mammary gland showed a great variability along time, displaying different trends in adolescence (36 and 51 days) and in adulthood (100 and 246 days of age). The *RASSF1A* gene expression showed a maximum at 100 days in all groups (except HCO) and a decrease thereafter (Fig 2a). The *TIMP3* gene expression decreased along time in all high fat groups, while reached its maximum at 100 days of age in LF group (Fig 2b). The high fat diets did not modify *RASSF1A* expression at the ages tested. *TIMP3* expression decreased in LF-HCO group compared to LF group at the end of the assay (246 days) (Fig 2b). In tumors, *RASSF1A* gene expression showed a significant decrease in both high olive oil groups in relation to LF and high corn oil groups (Fig 2c), while *TIMP3* expression was lower in LF-HOO group compared with the LF control (Fig 2d). Comparison between tissues showed a significant decrease in the expression of both genes in tumors in all experimental groups (Fig 2e and 2f).

### Effects of high fat diets and breast cancer on DNA promoter methylation of RASSF1A and TIMP3 genes

**Fig 3:**
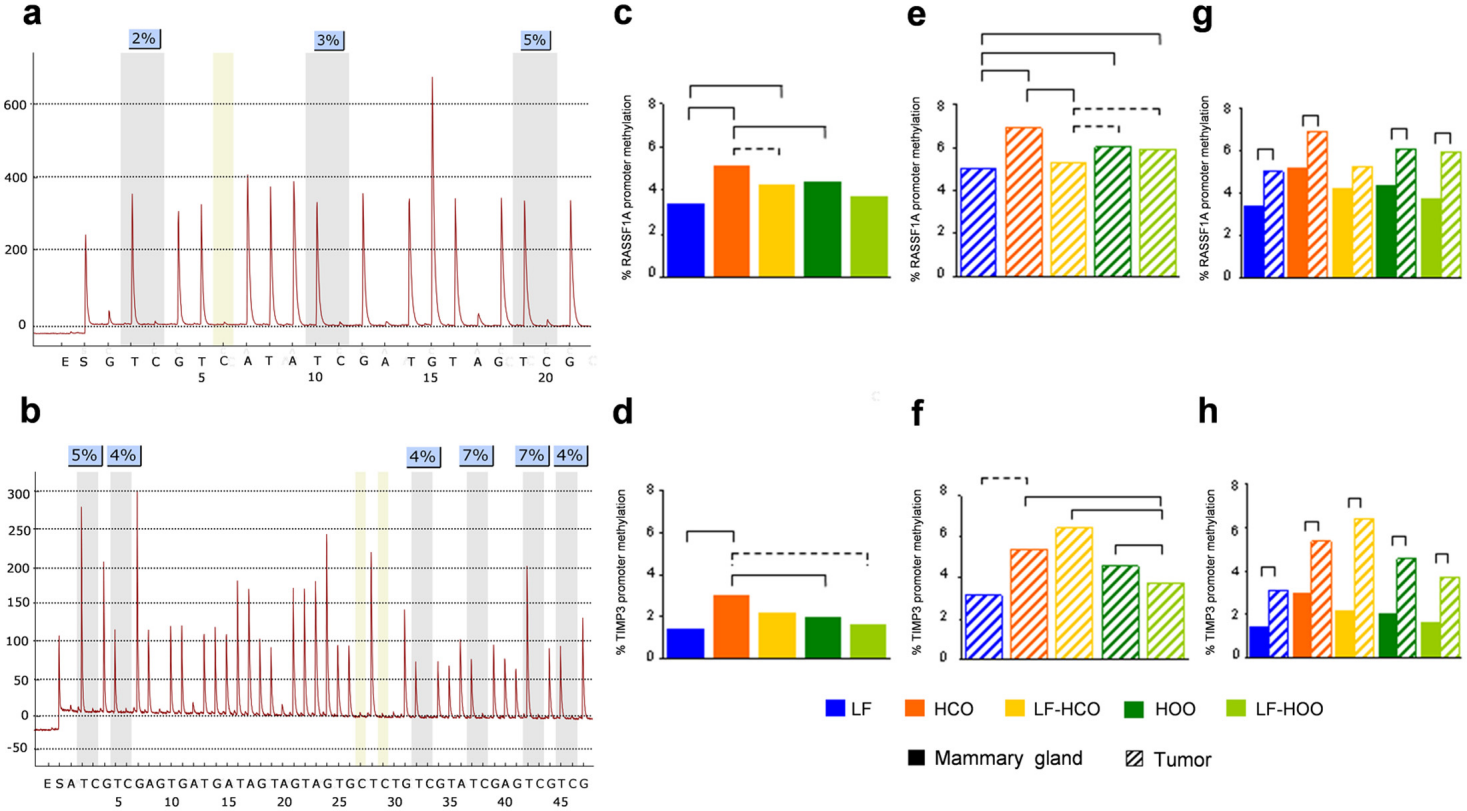
DNA promoter methylation of *RASSF1A* and *TIMP3* genes in mammary gland and tumor. Representative pyrogram of *RASSF1A* **(a)** and *TIMP3* **(b)** promoter methylation analyses. Determination of *RASSF1A* and *TIMP3* DNA methylation in mammary gland **(c, d)** and tumor **(e, f)** from all experimental groups at 246 days of age. Comparison of *RASSF1A* **(g)** and *TIMP3* **(h)** gene methylation between both tissues in each experimental group at 246 days of age. Data shown represent medians of the groups. Full lines connecting groups indicate differences statistically significant (p<0.05); dotted lines indicate differences close to significance (p<0.1).

The promoter methylation of *RASSF1A* (Fig 3a) and *TIMP3* (Fig 3b) genes were determined in bisulfite-modified DNAs from mammary glands and tumors at 246 days. In mammary gland, both groups fed the corn oil-enriched diet had significant increased values of *RASSF1A* methylation compared to LF group. Values from HCO were also significantly higher than those from HOO (Fig 3c). *TIMP3* mehtylation was also increased in HCO group compared to LF and high extra virgin olive oil groups (Fig 3d). In tumors, the methylation levels of *RASSF1A* increased in all high fat diet groups except in LF-HCO (Fig 3e). *TIMP3* promoter methylation tended to increase in HCO group versus the control, whilst the LF-HOO group had significantly lower levels compared with all other high fat groups (Fig 3f). Finally, comparison between tissues showed an increase in both *RASSF1A* and *TIMP3* promoter methylation in tumors, compared to mammary glands, in all the experimental groups. (Fig 3g and 3h).

### Effects of high fat diets and breast cancer on DNMT1, DNMT3a and DNMT3b mRNA relative levels

**Fig 4:**
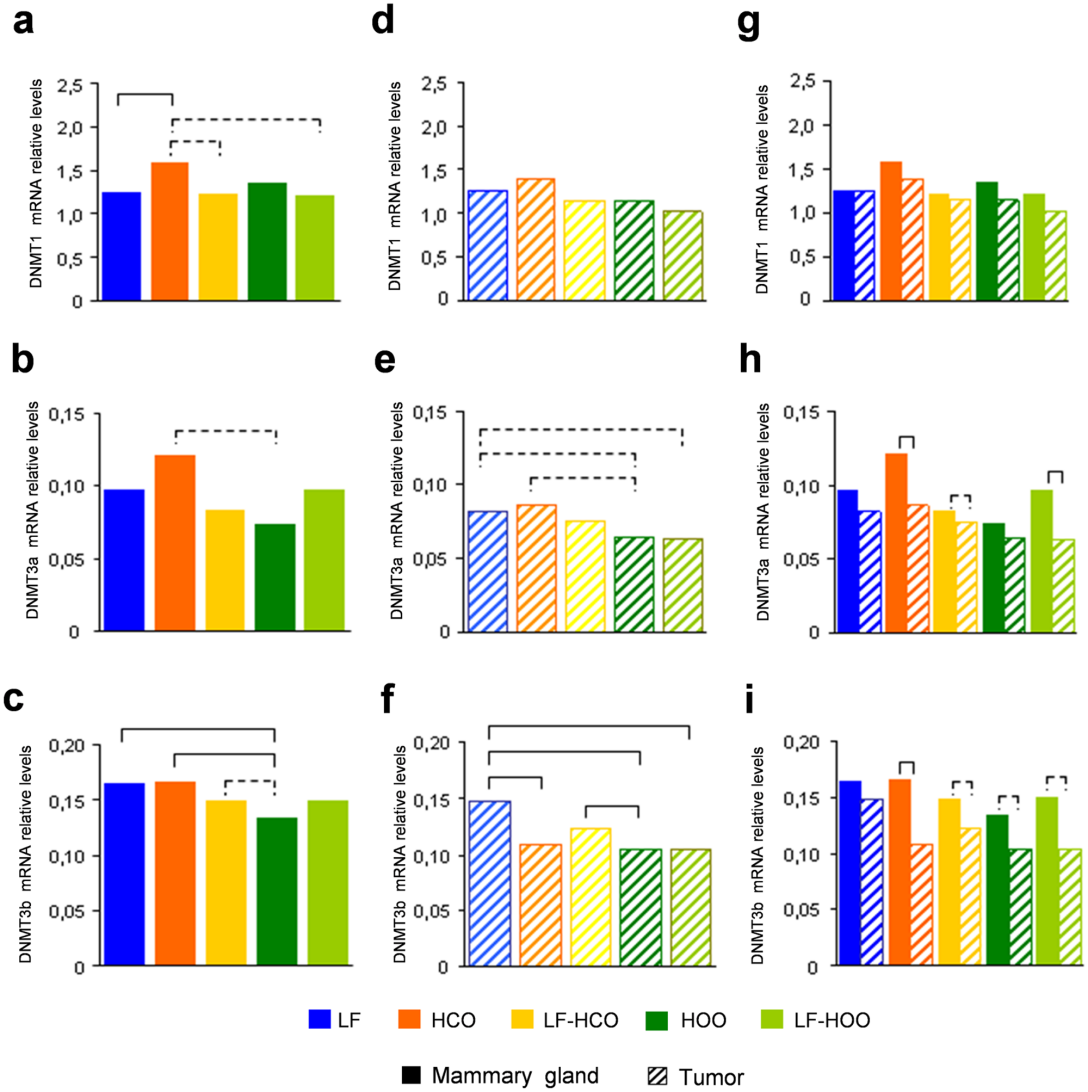
DNA methyltransferase gene expression in mammary gland and tumor. Determination of *DNMT1*, *DNMT3a* and *DNMT3b* mRNA relative levels in mammary gland **(a, b** and **c)** and tumors **(d, e** and **f)** from all experimental groups at 246 days of age. Comparison of *DNMT1* **(g)**, *DNMT3a* **(h)** and *DNMT3b* **(i)** mRNA relative levels between both tissues in each experimental group at 246 days of age. Data shown represent medians of the groups. Full lines connecting groups indicate differences statistically significant (p<0.05); dotted lines indicate differences close to significance (p<0.1).

The mRNA relative levels of the maintenance DNA methyltransferase (*DNMT1*) and the *de novo* methyltransferases (*DNMT3a* and *DNMT3b*) were analyzed by real time PCR in mammary gland and in tumors at the end of the assay. The *DNMT1* mRNA relative levels were always higher compared to *DNMT3a* and *DNMT3b* mRNA levels in both tissues and in all experimental groups (Fig 4). Expression levels of DNMT in mammary gland at 246 days of age showed a significant increase in *DNMT1* mRNA levels in HCO (Fig 4a) and a decrease in *DNMT3b* mRNA in HOO compared to LF and HCO groups (Fig 4c). In tumors, we did not find significant differences in *DNMT1* and *DNMT3a* mRNA relative levels among groups, although a trend to lower *DNMT3a* levels in EVOO groups was observed (Fig 4d and 4e). *DNMT3b* expression was decreased in HCO and in both high olive oil groups in comparison to the control (Fig 4f). We also compared values from mammary gland and tumor in each experimental group finding, in general, a decrease of DNMT expression in tumor, mainly in *DNMT3a* and *DNMT3b* (Fig 4 g–4i).

### Effects of high fat diets and breast cancer on DNMT activity

**Fig 5:**
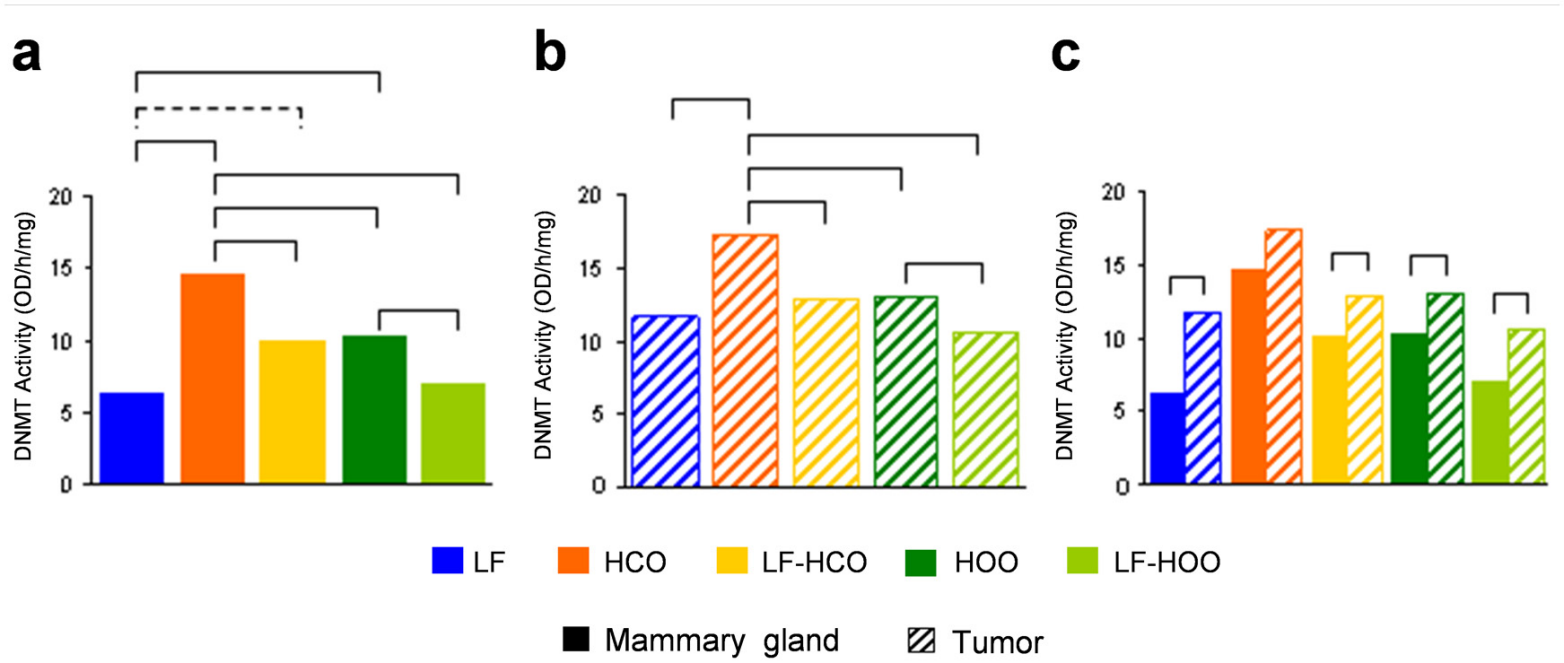
DNA methyltransferase activity in mammary gland and tumor. Determination of DNMT activity in mammary gland **(a)** and tumors **(b)** from all experimental groups and comparison between both tissues **(c)**. Data shown represent medians of the groups. Full lines connecting groups indicate differences statistically significant (p<0.05); dotted lines indicate differences close to significance (p<0.1).

We determined the total (*de novo* and maintenance) DNA methyltransferase activity at the end of the assay (246 days of age) in mammary gland and tumor. In both tissues, HCO group showed an increase in DNMT activity compared with all other groups. In addition, HOO group showed an increase in DNMT activity compared to LF-HOO group in both tissues and compared to low fat group in mammary gland (Fig 5a and 5b). Comparison between mammary gland and tumor in each experimental group showed a statistical significant increase in the tumor DNMT activity in all groups except in HCO (Fig 5c).

### Effects of high fat diets and breast cancer on histone modifications

**Fig 6:**
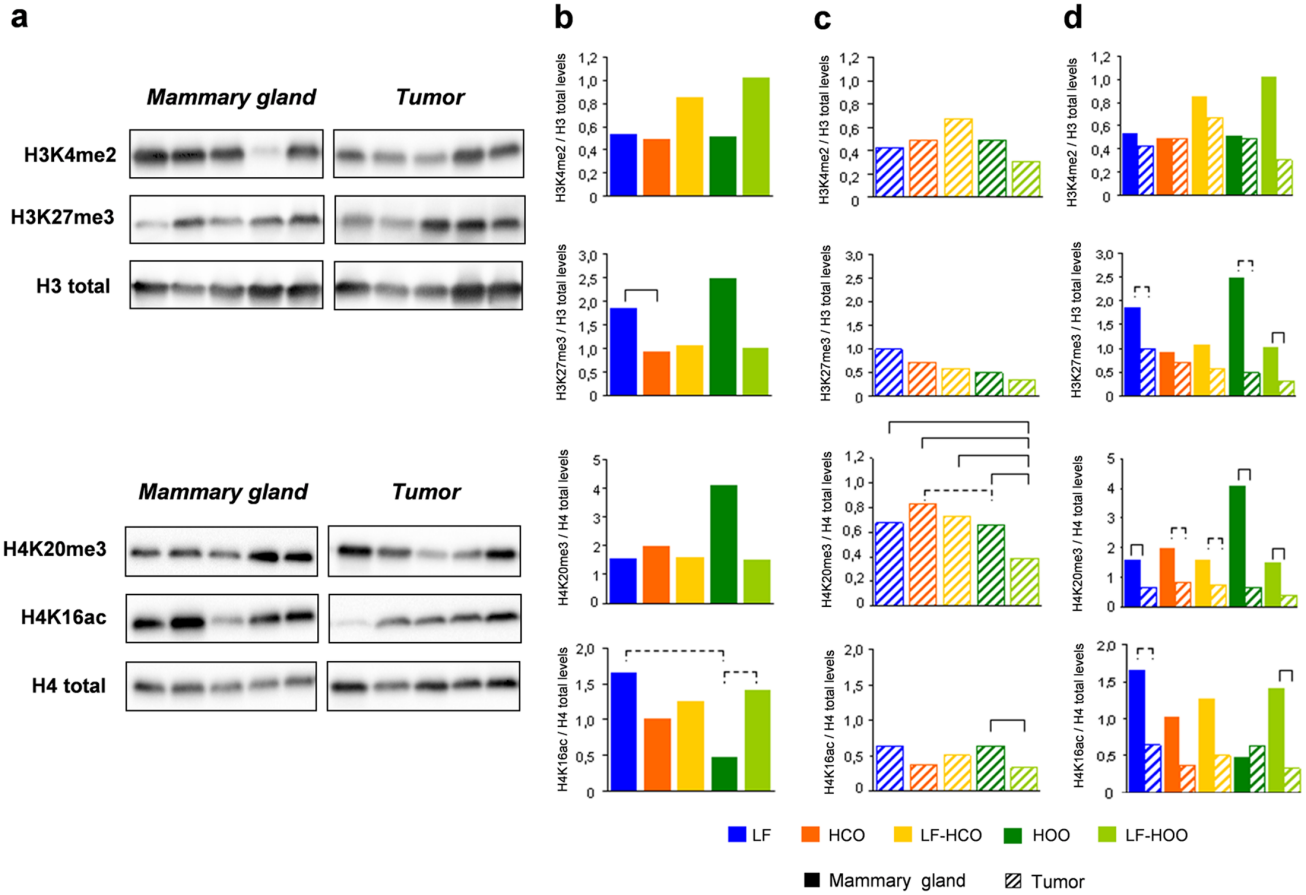
Global levels of histone 3 (H3K4me2, H3K27me3) and histone 4 (H4K20me3, H4K16ac) modifications in mammary gland and tumor. Representative images obtained for western blot data from mammary gland and tumor of histone 3 and histone 4 modifications **(a)**. Determination of relative levels of such modifications in mammary gland **(b)** and tumor **(c)** from all experimental groups at 246 days of age. Comparison of global levels of histone modifications between both tissues **(d)** in each experimental group. Data shown represent medians of the groups. Full lines connecting groups indicate differences statistically significant (p<0.05); dotted lines indicate differences close to significance (p<0.1).

Global levels of histone 3 (H3K4me2, H3K27me3) and histone 4 (H4K20me3, H4K16ac) modifications have been determined in mammary gland and tumor at 246 days of age by western blotting (Fig 6a). In mammary gland, we found no clear differences of such histone modifications among experimental groups, except a significant decrease of H3K27me3 relative levels in HCO group compared to LF group, and a trend to decrease the H4K16ac relative levels in HOO group (Fig 6b). In tumors, the high fat diets did not significantly influence the relative levels of histone 3 modifications (H3K4me2, H3K27me3). Results of histone 4 modifications showed a decrease of H4K20me3 relative levels in LF-HOO group compared with all other groups, and a decrease of H4K16ac in LF-HOO compared to HOO group (Fig 6c). We also compared the global levels of histone 3 and histone 4 modifications between mammary gland and tumor in each experimental group at 246 days of age finding, in general, a decrease of such modifications in tumor (Fig 6d).

## Discussion

Disruptions of epigenetic patterns including global DNA hypomethylation, tumor suppressor gene promoter hypermethylation and aberrant histone modifications have been considered hallmarks of cancer [20]. These epigenetic changes are reversible and may be modified by nutritional factors, including dietary lipids. In the present study, we have investigated the effect of diets rich in corn oil or in extra virgin olive oil on DNA methylation and histone modifications in the rat DMBA-induced breast cancer model. Our results revealed different mechanisms of action of such diets on epigenetic patterns, which could play an important role in the strong enhancing effect described for the high corn oil diet and the weaker effect of the EVOO diet on breast cancer progression.

Previous results in the same animals that have been used in this work, showed a strong tumor enhancing effect of the high corn oil diet when administered from weaning (Group HCO) and from induction (Group LF-HCO), while a weak effect of the high olive oil diet administered in the same periods [21, 22]. Thus, by 246 days of age the high corn oil diet induced a significantly higher percentage of tumor-bearing animals (incidence: LF 80% -16/20-, HCO 100% -20/20-, LF-HCO 100% -20/20-, HOO 75% -15/20-, LF-HOO 85% -17/20-) and tumor yield (number of tumors: LF 46, HCO 99, LF-HCO 87, HOO 58, LF-HOO 83). Moreover, tumors from groups fed such diet tended to appear earlier and had higher volume, and displayed morphological characteristics of higher malignancy. Hence, in this work we aimed to get insight into the molecular mechanisms by which these high fat diets had their differential effects on experimental mammary carcinogenesis.

Global DNA hypomethylation has been associated with neoplastic transformation, tumorigenesis, cancer progression and chromosomal instability [7]. To study the effect of high fat diets on genomic methylation, we have determined the global DNA methylation level of mammary gland and tumor in rats fed diets rich in corn oil or in EVOO. We first observed variations along time in the genomic DNA methylation of the mammary gland, displaying decreases at puberty (from 23 to 36 days) that may be related to the mammary gland development [23, 24]. We have also found an age-related decrease of methylation in adulthood (from 100 to 246 days). Although no data have been reported in this model, such result is in accordance with published evidence in human tissues, which demonstrate that physiologic aging is also accompanied by widespread epigenetic changes, including gradual loss of global DNA methylation [25]. In relation to the effect of diet, our data also showed higher levels of methylation in EVOO groups, both in mammary gland at all ages and in tumor, suggesting an inhibitory effect of EVOO on cancer-related global DNA hypomethylation. This result can be related to previous experimental series where we have observed from a weak protective effect of the high EVOO diet [16] to a weak enhancing effect after chronic intake [21, 22]. Finally, we have also compared the levels of genomic methylation among mammary gland and tumor in each experimental group at 246 days of age. Contrary to our expectations [20], levels in tumor were not lower than in mammary gland. This could be related to the fact that this gland has suffered a carcinogenic insult. Although no data has been found in DMBA-induced mammary gland, in ACI rats exposure to estrogen and ionizing irradiation decrease global DNA methylation of this tissue [26, 27].

It is well-known that global DNA hypomethylation and tumor suppressor hypermethylation promote carcinogenesis, affecting DNA stability and gene expression [20, 28]. To further investigate the effects of diets, we have analyzed the expression of the tumor suppressor genes *RASSF1A* and *TIMP3*, which have important roles on the key hallmarks that a neoplastic cell acquires, and are frequently down-regulated in breast cancer [29, 30]. We have determined the mRNA relative levels of both genes in mammary gland at all ages and in tumor. We have observed changes in mammary gland along lifetime, with *RASSF1A* and *TIMP3* mRNA levels increasing during the adolescence (36 and 51 days). This regulation may be related to the proliferation and differentiation associated with the mammary gland development, and is in concordance with the reported increase in tumor suppressor expression at the end of puberty in such gland [31]. We also found a decrease in the *RASSF1A* and *TIMP3* gene expression in the adulthood along time (100 and 246 days of age), in accordance with promoter methylation and transcriptional silencing of tumor suppressor genes with aging [32]. In relation to the effects of the high fat diets, little influence was observed in mammary gland, whilst in tumor the EVOO groups showed lower levels of *RASSF1A* and *TIMP3* mRNA. As expected, comparison between tissues indicated a down-regulation in tumor versus mammary gland in all experimental groups, suggesting an inactivation of these tumor suppressor genes in cancer [33].

Hypermethylation of CpG islands in the promoter regions of tumor suppressor genes is one of the main mechanisms for gene silencing [34]. Thus, we have analyzed the promoter methylation of *RASSF1A* and *TIMP3* genes in mammary gland and tumor at 246 days of age, finding a significant increase in both tissues in the corn oil-enriched diet groups, especially in HCO. On the other hand, the EVOO diet, despite being also high fat, only increased *RASSF1A* promoter methylation in the tumor, suggesting a selective mechanism of action on promoter methylation of such diet. In this sense, hydroxytyrosol and oleuropein, the most representative poliphenols in extra virgin olive oil, are able to selectively regulate Type 1 cannabinoid receptor (CB1) expression, a tumor suppressor gene, in Caco-2 cells and in the rat colon via epigenetic mechanisms [35]. In our study, the comparison between tissues showed an increase of *RASSF1A* and *TIMP3* methylation in tumor in all experimental groups. It is in concordance with the hypermethylation of tumor suppressor genes described in cancer [29, 30].

DNA methylation is catalyzed by the enzyme 5-cytosine DNA methyltransferase (DNMT). There are three main DNMT enzymes that possess catalytic methyltransferase activity: *DNMT1* (maintenance methyltransferase), *DNMT3a* and *DNMT3b* (*de novo* methyltransferases). Hence, we investigated the effects of the high fat diets on DNMT function, determining mRNA levels of *DNMT1*, *DNMT3a* and *DNMT3b*, as well as DNMT activity in mammary gland and tumor at 246 days of age. We found higher *DNMT1* mRNA levels compared to *de novo* DNMT in both tissues and in all experimental groups, according to the literature [10], and a coordinated expression of *DNMT1*, *DNMT3a* and *DNMT3b* in mammary gland and tumor, as it has been described in several tissues [36]. In relation to the effect of dietary lipids, in mammary gland we observed an increase in *DNMT1* expression in HCO and a decrease in *DNMT3b* expression in HOO group. In tumor, *DNMT3b* mRNA was decreased in all high fat groups. Surprisingly, a decrease of DNMT expression in tumor, compared to mammary gland was, also detected, mainly in *DNMT3a* and *DNMT3b*. Thus, we analyzed DNMT activity finding that, as expected [37], it was increased in tumors versus mammary gland. This imbalance between mRNA levels and DNMT activity indicates that DNMT function is relevantly regulated on the post-transcriptional level. Furthermore, when we analyzed the DNMT activity in relation to the diet administered, an increase in HCO group was found in both tissues (mammary gland and tumor). A consequence of this increase in HCO group could be the higher values of DNA promoter methylation of *RASSF1A* and *TIMP3* genes detected in both tissues [38, 39], which could be one of the mechanisms of the stimulating effect of carcinogenesis of such diet [3, 4, 16, 24, 40, 41]. On the other hand, the olive oil-enriched diets showed intermediate values between the obtained in the low fat group and the corn oil-enriched diet groups, maybe due to the action of poliphenols of EVOO which have shown DNMT inhibition properties [42–44]. Finally, in both tissues there was an increase in DNMT activity in HCO and HOO groups compared with their counterparts LF-HCO and LF-HOO, suggesting that time and duration of high fat diet implementation could have an effect on DNMT activity in such tissues, promoting the carcinogenesis process.

DNA methylation and histone modifications interact with each other in the regulation of gene expression [20]. Therefore, we analyzed the effects of the high fat diets on global levels of histone 3 (H3K4me2, H3K27me3) and histone 4 (H4K20me3, H4K16ac) modifications, in mammary gland and tumor at 246 days of age. As far as we know, no data has been published in rat DMBA-induced breast cancer model about dietary effects on these histone modifications. Results showed a decrease in mammary gland of H3K27me3 relative levels in HCO group, and a decrease in tumor of H4K20me3 relative levels in LF-HOO group compared with all other groups. These results could be considered a mechanism by which these lipids influence rat carcinogenesis [3, 4]. Finally, we also compared the global levels of histone 3 and histone 4 modifications between mammary gland and tumor, finding, in general, higher levels of such modifications in mammary gland tissue, according to published data [11–13].

In conclusion, the corn oil-enriched diet increased DNMT activity both in mammary gland and tumor, more likely as a consequence of the stimulation of activity than of the increase in mRNA levels. This effect was reflected in the promoter methylation of *RASSF1A* and *TIMP3* genes in both tissues. However, methylation changes of these genes did not reflect the variations observed in the mRNA levels, indicating the relevant role of other mechanisms regulating their transcriptional silencing. In any case, increases in DNMT activity are related to gene-specific methylation, suggesting that the corn oil-enriched diet may induce hypermethylation of several genes, whose silencing would have a role on the carcinogenesis-promoting effect of this diet [9]. *RASSF1A* and *TIMP3* are tumor suppressor genes that could be inactivated though epigenetic mechanisms in early-events of the carcinogenesis process and, therefore, in the long-term their expression may not reflect the degree of malignancy of tumors. Respect the olive oil-enriched diet, on the one hand, decreased the levels of global DNA hypomethylation and, on the other, changed histone modification patterns. We have previously demonstrated a strong enhancing effect of high corn oil diet on mammary carcinogenesis, while the EVOO diet, despite being high fat, had a weaker effect. Considering the unspecific promoter influence that all high fat diets have on experimental carcinogenesis [45], the EVOO must have some beneficial effects that may partially counteract the total fat intake. Its richness in MUFA and other bioactive components such as phenolic compounds, squalene could be the responsible of the weak protective effect detected in our study. Altogether, our results suggest different effects of the high fat diets, rich in corn oil or in olive oil, on epigenetic mechanisms with a relevant role in the neoplastic transformation. This specific influence could be at the basis, at least in part, of the differential promoter effects described by PUFA n-6 enriched oils and EVOO on breast cancer progression.

## Supporting Information

S1 Fig Experimental design. (TIF)

S1 File Detailed data for each variable. (XLS)

## Abbreviations

DMBA: dimethylbenz[α]anthracene
DNMT: DNA methyltransferase
EVOO: extra virgin olive oil
HCO: high corn oil
HOO: high olive oil
LF: low fat
LF-HCO: low fat-high corn oil
LF-HOO: low fat-high olive oil
MUFA: monounsturated fatty acids
PUFA: polyunsaturated fatty acids
