# Supplementary figures and images for: The Role of Dietary Extra Virgin Olive Oil and Corn Oil on the Alteration of Epigenetic Patterns in the Rat DMBA-Induced Breast Cancer Model
Source: PLoS One. 2015 Sep 24;10(9):e0138980. doi: 10.1371/journal.pone.0138980 (PMC4581736; doi:10.1371/journal.pone.0138980)

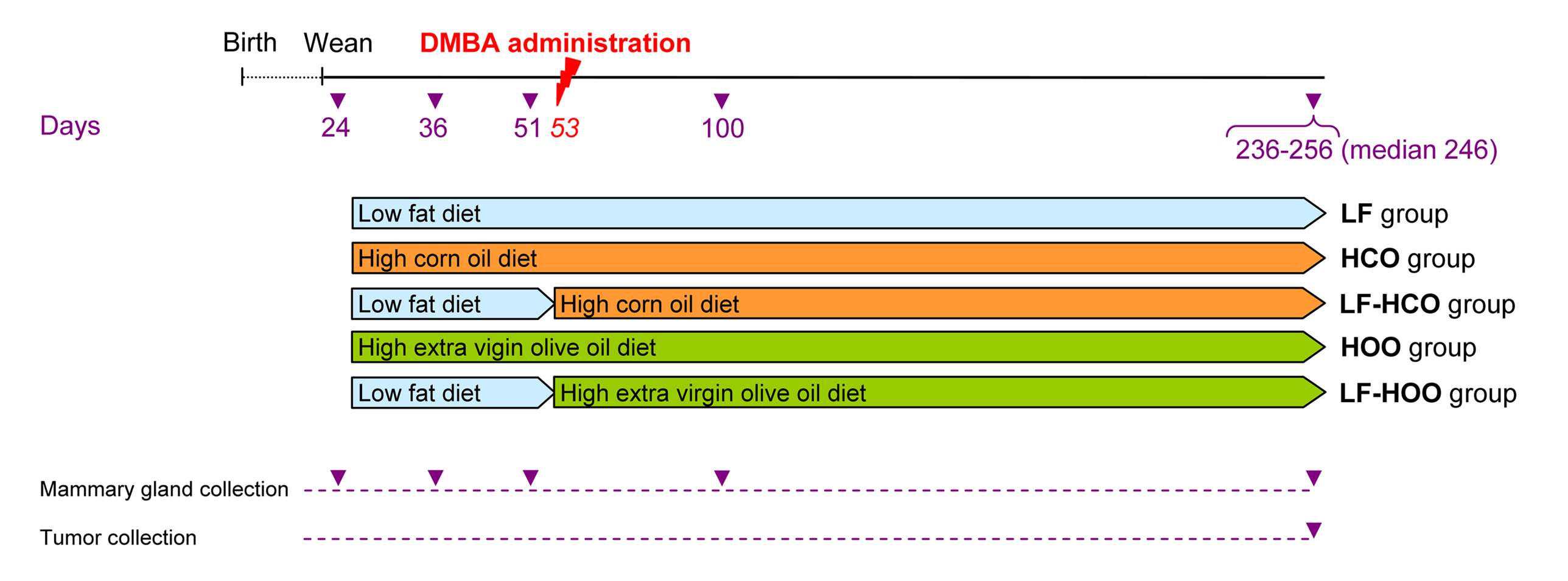

Supplement: S1 Fig — (TIF) [file pone.0138980.s001.tif]
